# Tobacco Use Cessation Among Quitline Callers Who Implemented Complete Home Smoking Bans During the Quitting Process

**DOI:** 10.5888/pcd14.170139

**Published:** 2017-10-26

**Authors:** Alesia M. Jung, Nicholas Schweers, Melanie L. Bell, Uma Nair, Nicole P. Yuan

**Affiliations:** 1 Department of Epidemiology and Biostatistics, Mel and Enid Zuckerman College of Public Health, University of Arizona, Tucson, Arizona; 2 School of Psychology, University of Sydney, Sydney, Australia; 3 Department of Health Promotion Sciences, Mel and Enid Zuckerman College of Public Health, University of Arizona, Tucson, Arizona

## Abstract

**Introduction:**

The implementation of a home smoking ban (HSB) is associated with tobacco use cessation. We identified which quitline callers were most likely to report 30-day cessation among those who implemented complete HSBs after enrollment.

**Methods:**

Our sample consisted of callers to the Arizona Smokers’ Helpline who enrolled from January 1, 2011, through July 26, 2015, and who reported no HSB at enrollment and a complete HSB by 7-month follow-up. We used logistic regression to estimate associations between no use of tobacco in the previous 30 days (30-day quit) at 7-month follow-up and demographic characteristics, health conditions, tobacco use, and cessation strategies.

**Results:**

At 7-month follow-up, 65.4% of 399 callers who implemented a complete HSB reported 30-day quit. Lower odds of tobacco use cessation were associated with having a chronic health condition (odds ratio [OR], 0.31; 95% confidence interval [CI], 0.18–0.56) and living with other smokers (OR, 0.46; 95% CI, 0.29–0.73). Higher odds of tobacco cessation were associated with completing 5 or more telephone coaching sessions (OR, 2.48; 95% CI, 1.54–3.98) and having confidence to quit (OR, 2.05; 95% CI, 1.05–3.99). However, confidence to quit was not significant in the sensitivity analysis.

**Conclusion:**

Implementing an HSB after enrolling in quitline services increases the likelihood of cessation among some tobacco users. Individuals with complete HSBs were more likely to quit if they did not have a chronic health condition, did not live with another smoker, and were actively engaged in coaching services. These findings may be used by quitlines to develop HSB intervention protocols primarily targeting tobacco users most likely to benefit from them.

## Introduction

Home smoking bans (HSBs) are household rules that restrict smoking from certain areas (partial HSB) or all areas (complete HSB) (1). Implementing HSBs may facilitate changes in smoking behavior by limiting exposure to smoking cues from household members and visitors who smoke (2). Implementing any type of HSB is associated with tobacco use cessation (2–4). However, complete HSBs are a more effective cessation strategy than partial HSBs (5), which present challenges in enforcing smoking restrictions (6).

In the United States, quitlines provide evidence-based cessation services to residents of all 50 states, the District of Columbia, and Puerto Rico, reaching diverse populations, including those from underserved and vulnerable communities (7,8). Because success rates of cessation strategies vary among individuals (9), quitlines seek to expand the diversity of cessation services and tailor them to specific groups of smokers to optimize service delivery and improve quit rates (10). One area that has received little attention is the use of HSB interventions by quitlines. Identifying callers who are most likely to benefit from HSB interventions and quit tobacco use may inform the development of quitline protocols for HSB interventions for specific groups of tobacco users. The objective of our study was to describe predictors of tobacco use cessation among a sample of adults who implemented a complete HSB after enrolling in services from the Arizona Smokers’ Helpline (ASHLine).

## Methods

This retrospective cohort study was based on data from ASHLine, which is a state-funded quitline that supports cessation among tobacco users who live in Arizona (www.ashline.org). Callers who complete telephone assessments at enrollment are given the opportunity to work with a trained cessation coach who assists them through the process of quitting. Informed by motivational interviewing and evidence-based cognitive behavioral strategies, coaches provide up to 3 months of weekly telephone counseling. Callers are provided information and guidance on self-regulation, identification of triggers, stimulus-management and urge-management strategies, positive reinforcement, quit smoking tips, preparation for setting a quit day, and relapse prevention. Eligible callers are also provided with up to 4 weeks of free nicotine replacement therapy. Because the study used de-identified caller data, the study protocol was deemed exempt by the University of Arizona’s institutional review board.

### Eligible participants

Participants were male and female adult callers (aged 18 y or older) who enrolled in ASHLine from January 1, 2011, through July 26, 2015. Assessments were conducted at enrollment and 7-month follow-up by using telephone surveys administered by ASHLine staff members. To focus the study on the most effective type of HSB, a complete (rather than a partial) HSB, we included participants in the analysis only if they implemented a complete HSB between enrollment and 7-month follow-up. HSB status was determined by asking, “Is smoking allowed in your home?” Responses included “smoking not allowed,” “smoking allowed in some places,” or “smoking allowed anywhere.” Implementation of a complete HSB was defined as having a response of “smoking allowed anywhere” at enrollment to indicate that no HSB was in place and a response of “smoking not allowed” at the 7-month follow-up assessment. Callers were excluded from the study if they did not report implementing a complete HSB by the 7-month follow-up.

### Outcome and other variables

The study outcome was smoking cessation, which was determined at the 7-month follow-up assessment. Participants were asked the question “Have you used tobacco products in the last 30 days?” (30-day quit). Callers who responded no to the question at 7-month follow-up were categorized as quitters, and callers who responded yes were categorized as nonquitters. Smoking cessation was chosen as the study outcome because it is one of the primary measures of quitline effectiveness (11). Callers missing a response to this question were excluded from analysis. Several variables were assessed during enrollment, including demographic characteristics, health conditions, and tobacco use. Demographic characteristics were caller’s age (years); sex (male, female); race (white, black, Asian, American Indian, or multiracial/other); Hispanic or Latino ethnicity; insurance type (private insurance, Arizona’s Medicaid [Arizona Health Care Cost Containment System], or uninsured); and education level (high school diploma or no diploma). Race was categorized as white, black, or other. Missing responses for Hispanic or Latino ethnicity were imputed to be no. Insurance type was used as a proxy measurement for socioeconomic status and was categorized as private insurance or not insured/underinsured. The presence of children in the household was determined by asking “Do you have children under the age of 18 living in your household?” We ascertained the age of the youngest child in the household. The presence of other smokers in the household was evaluated by asking “Do others smoke at home?” and dichotomized as yes or no.

Having a chronic or mental health condition was self-reported at the time of enrollment. Individuals were categorized as having a chronic health condition if they had at least one of the following: asthma, hypertension, cancer, chronic obstructive pulmonary disease, diabetes, or heart disease. Presence of a mental health condition was ascertained by asking if they had ever been treated for “mental health or emotional challenges, such as anxiety disorder, depression, bipolar disorder, alcohol or drug abuse, or schizophrenia.” Responses included yes, no, or “I don’t know.” Callers could also refuse to answer these questions. Refusals were considered missing data, and responses of “I don’t know” were inferred to be no.

Variables describing tobacco use were also assessed at enrollment. The variables included age of initiation (years); nicotine dependence as measured by the Fagerström Test for Nicotine Dependence score (ranging from 0–10), with higher scores indicating greater dependence (12); and confidence to quit for 24 hours, dichotomized as “confident” (caller’s response of “confident,” “very confident” or “extremely confident”) and “not confident” (responses of “somewhat confident” or “not confident”).

Variables included in the analysis that described cessation strategies were use of medication for tobacco use cessation and the number of coaching sessions completed by the caller. Self-reported use of any medication for tobacco use cessation (eg, nicotine replacement therapy or medications such as Zyban [GlaxoSmithKline] or Chantix [Pfizer]) was categorized as either yes or no. Data on the number of coaching sessions a caller completed between enrollment and 7-month follow-up were obtained from ASHLine records. Number of coaching sessions was considered both as a continuous variable and a binary variable, zero to 4 coaching sessions and 5 or more coaching sessions, as recommended by the North American Quitline Consortium best practice protocols (13).

### Statistical analyses

Logistic regression was used to estimate associations between 30-day quit and demographic characteristics, health conditions, tobacco use, and cessation strategy variables. Covariates included in the model were prespecified and based on theoretical relevance. All reported odds ratios (ORs) and 95% confidence intervals (CIs) were adjusted for all covariates in the model. Sensitivity analysis was performed by using multiple imputation with chained equations to assess the sensitivity of our results to missing covariates (14); only covariates specified in the analytical model and the outcome were used for this step. The imputation models contained all the covariates from the analytical model, the smoking cessation outcome, covariates associated with missingness, and auxiliary covariates found to be associated (Pearson correlation coefficient >0.20) with predictors that were being imputed. Twenty complete data sets were imputed and analyzed with logistic regression, and estimates were combined by using Rubin’s Rules (15).

Linearity in the logit for continuous variables was tested by using restricted cubic splines (16). If linearity in the logit was not met for a continuous covariate, the covariate was categorized to meet the assumption for logistic regression. Linearity in the logit was met for age at enrollment, age of tobacco use initiation, and tobacco dependence. Number of coaching sessions did not meet this assumption and was therefore categorized as zero to 4 sessions and 5 or more sessions.

All statistical tests used a significance level of .05 and were performed using SAS 9.4 (SAS Institute, Inc).

## Results

Of the original 49,284 callers, 38,948 callers with a complete or partial HSB at enrollment or missing data on HSB status were excluded from analysis (Figure). Of the remaining 10,336 callers, 9,587 were excluded because they were missing data on 30-day quit status (n = 5,865) or they reported not implementing a complete HSB or were missing data on home smoking ban (n = 3,722) at 7-month follow-up. The remaining 749 callers had 7-month quit information; 350 of these callers were excluded because they had missing data on covariates. We found missing values for almost all covariates; the covariates with the highest percentage of missing values were race (11.2%) and use of medication for tobacco use cessation (21.9%). This left 399 callers for the primary analysis; of these, 261 (65.4%) were quitters and 138 (34.6%) were nonquitters.

**Figure:**
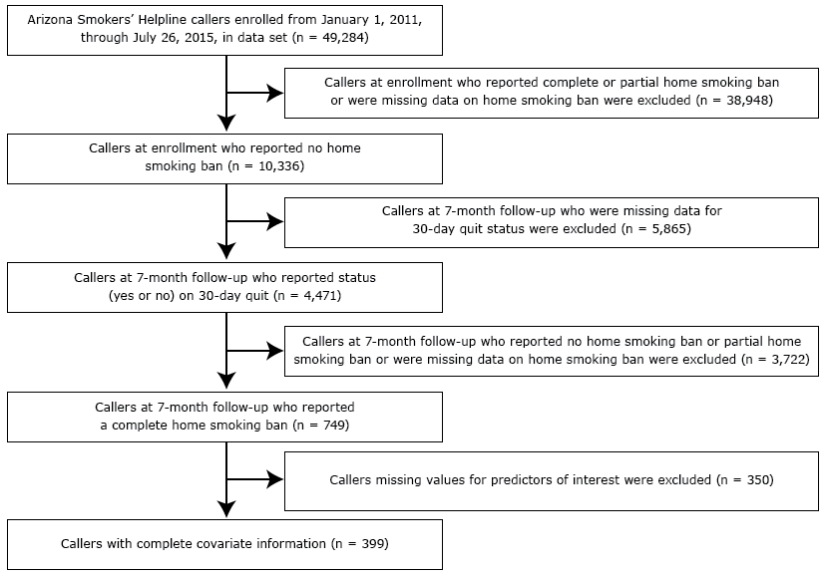
Selection of callers who enrolled in the Arizona Smokers’ Helpline (ASHLine) and were included in analysis of home smoking bans, Arizona, January 1, 2011, through July 26, 2015. Thirty-day quit was defined as callers who said they had not used tobacco products in the last 30 days at 7-month follow-up.

Callers ranged in age from 19 to 89 years, and most callers were white, were non-Hispanic, had at least a high school diploma, and had no children under the age of 18 at home (Table 1). The distribution of covariates was similar between quitters and nonquitters, with a few significant exceptions. Nonquitters were more likely than quitters to live with another smoker in the home (53.1% vs 39.2%) and have at least 1 chronic condition (73.3% vs 64.6%). Quitters were more likely than nonquitters to report using medication for tobacco use cessation (67.0% vs 45.9%) and completing more coaching sessions (median of 7 sessions vs median of 3 sessions). Although the difference was not significant, we observed that nonquitters were more likely than quitters to be white (76.2% vs 68.4%) and to be uninsured (23.1% vs 20.3%).

**Table 1:** Enrollment Characteristics of ASHLine Study Population Enrolled From January 1, 2011, Through July 26, 2015, Who Implemented a Complete Home Smoking Ban (n = 749), Stratified By 7-Month Quit Status^a^

Variable	Nonquitters	Quitters
**All, n (%)**	277 (37.0)	472 (63.0)
**Sex, n (%)**		
Male	111 (40.1)	196 (41.5)
Female	164 (59.2)	273 (57.8)
Missing data	2 (0.7)	3 (0.6)
**Age, y**		
Median (IQR)	54 (47–62)	58 (50–64)
Missing data, n (%)	0	3 (0.6)
**Race, n (%)**		
White	211 (76.2)	323 (68.4)
Black	28 (10.1)	55 (11.7)
Asian	2 (0.7)	2 (0.4)
American Indian	3 (1.1)	12 (2.5)
Multiracial/Other	8 (2.9)	21 (4.5)
Missing data	25 (9.0)	59 (12.5)
**Hispanic or Latino, n (%)**	20 (7.2)	65 (13.8)
**Insurance type, n (%)**		
Private	132 (47.7)	258 (54.7)
AHCCCS	80 (28.9)	114 (24.2)
None	64 (23.1)	96 (20.3)
Missing data	1 (0.4)	4 (0.9)
**Has high school diploma, n (%)**		
Yes	217 (78.3)	379 (80.3)
No	38 (13.7)	78 (16.5)
Missing data	22 (7.9)	15 (3.2)
**Age of youngest child in household, n (%), y**		
No children <18	230 (83.0)	406 (86.0)
<1	1 (0.4)	1 (0.2)
1–4	8 (2.9)	15 (3.2)
5–11	15 (5.4)	15 (3.2)
12–17	13 (4.7)	21 (4.5)
Missing data	10 (3.6)	14 (3.0)
**Other smokers reside in household, n (%)**		
Yes	147 (53.1)	185 (39.2)
No	118 (42.6)	265 (56.1)
Missing data	12 (4.3)	22 (4.7)
**Nicotine dependence^b^**		
Median (IQR) score	6 (4–7)	5 (4–7)
Missing data, n (%)	3 (1.1)	43 (9.1)
**Age of tobacco use initiation, y**		
Median (IQR)	16 (14–18)	16 (14–19)
Missing data, n (%)	19 (6.8)	37 (7.8)
**Has confidence to quit for 24 hours^c^, n (%)**		
Yes	227 (82.0)	380 (80.5)
No	37 (13.4)	74 (15.7)
Missing data	13 (4.7)	18 (3.8)
**Has at least 1 chronic health condition^d^, n (%)**		
Yes	203 (73.3)	305 (64.6)
No	74 (26.7)	167 (35.4)
**Has a mental health condition^e^, n (%)**		
Yes	111 (40.1)	186 (39.4)
No	163 (58.8)	283 (60.0)
Missing data	3 (1.1)	3 (0.6)
**Uses medication for tobacco use cessation, n (%)**		
Yes	127 (45.9)	316 (67.0)
No	50 (18.0)	92 (19.5)
Missing data	100 (36.1)	64 (13.6)
**Number of telephone coaching sessions before 7-month follow-up, median (IQR)**	3 (1–6)	7 (3–10)

Abbreviations: AHCCCS, Arizona Health Care Cost Containment System; ASHLine, Arizona Smokers’ Helpline; IQR, interquartile range.

a Percentages may not sum to 100 due to rounding.

b Measured by the Fagerström Test for Nicotine Dependence score (ranging from 0–10), with higher scores indicating greater dependence (12).

c Dichotomized as “confident” (caller’s response of “confident,” “very confident,” or “extremely confident”) and “not confident” (responses of “somewhat confident” or “not confident”).

d One or more of the following: asthma, hypertension, cancer, chronic obstructive pulmonary disease, diabetes, or heart disease.

e Treated for any of the following conditions: mental health or emotional challenges, such as anxiety disorder, depression, bipolar disorder, alcohol or drug abuse, or schizophrenia.

The adjusted odds of tobacco use cessation at 7-month follow-up were lower for callers who reported living with other smokers in the home (OR, 0.46; 95% CI, 0.29–0.73) or having at least 1 chronic health condition (OR, 0.31; 95% CI, 0.18–0.56). Callers were more likely to quit if they were confident in their ability to quit (OR, 2.05; 95% CI, 1.05–3.99) or if they completed 5 or more coaching sessions (OR, 2.48; 95% CI, 1.54–3.98) (Table 2).

**Table 2:** Odds Ratios of 30-Day Quit^a^, by Demographic, Tobacco Use History, Tobacco Dependence, and Cessation Strategy Covariates Among ASHLine Callers Who Implemented Complete Home Smoking Bans (n = 399), Arizona, January 1, 2011, Through July 26, 2015

Variable	Odds Ratio (95% Confidence Interval)^b^
**Sex**	
Male	1.09 (0.68–1.74)
Female	1 [Reference]
**Age (per 5-y increase in age)^c^**	1.11 (0.99–1.24)
**Race**	
White	1 [Reference]
Black	1.35 (0.63–2.95)
Other	1.56 (0.61–3.99)
**Hispanic or Latino**	
Yes	1.17 (0.29–4.68)
No	1 [Reference]
**Insurance type**	
Private	1 [Reference]
Not insured or AHCCCS	0.90 (0.56–1.46)
**Has high school diploma, n (%)**	
No	1 [Reference]
Yes	0.65 (0.31–1.38)
**Child (<18 y) resides in household**	
Yes	1.08 (0.48–2.46)
No	1 [Reference]
**Other smokers reside in household**	
Yes	0.46 (0.29–0.73)
No	1 [Reference]
**Nicotine dependence (per 1-point increase in score)^d^**	0.92 (0.83–1.02)
**Age of tobacco use initiation (per 5-y increase in age)^e^**	1.03 (0.83–1.27)
**Has confidence to quit for 24 hours^f^**	
Yes	2.05 (1.05–3.99)
No	1 [Reference]
**Has at least 1 chronic health condition^g^**	
Yes	0.31 (0.18–0.56)
No	1 [Reference]
**Has a mental health condition^h^**	
Yes	1.28 (0.78–2.11)
No	1 [Reference]
**Uses medication for tobacco use cessation**	
Yes	1.20 (0.71–2.03)
No	1 [Reference]
**Number of telephone coaching sessions before 7-month follow-up**	
0–4	1 [Reference]
≥5	2.48 (1.54–3.98)

Abbreviations: AHCCCS, Arizona Health Care Cost Containment System; ASHLine, Arizona Smokers’ Helpline.

a Defined as no use of tobacco in the previous 30 days, reported at 7-month follow-up.

b Odds ratios have been adjusted for all other covariates in the model.

c Odds ratios were calculated for an increment of 5 years in age. That is, for every 5-year increase in age, the odds of quitting tobacco for those who implemented a complete home smoking ban was 1.11 times the odds of those who had not implemented a ban.

d Measured by the Fagerström Test for Nicotine Dependence score (ranging from 0–10), with higher scores indicating greater dependence (12). Odds ratios were calculated for a 1-point increase in the Fagerström Test score.

e Odds ratios were calculated for an increment of 5 years in age.

f Dichotomized as “confident” (caller’s response of “confident,” “very confident,” or “extremely confident”) and “not confident” (responses of “somewhat confident” or “not confident”).

g One or more of the following: asthma, hypertension, cancer, chronic obstructive pulmonary disease, diabetes, or heart disease.

h Treated for any of the following issues: mental health or emotional challenges, such as anxiety disorder, depression, bipolar disorder, alcohol or drug abuse, or schizophrenia.

Using the multiply imputed data for missing values, the results of the primary analysis changed for several covariates (Table 3). The effects of living with other smokers and having at least 1 chronic condition were attenuated but still significant. The association for confidence to quit was no longer significant in the sensitivity analysis. The association between completing 5 or more coaching sessions and quitting smoking increased from an OR of 2.48 (95% CI, 1.54–3.98) to an OR of 3.25 (95% CI, 2.33–4.55).

**Table 3:** Results of Sensitivity Analysis: Odds Ratios (ORs) of 30-Day Quit^a^ for Demographics, Tobacco Use History, Tobacco Dependence and Cessation Strategy Covariates Among ASHLine Callers Who Implemented a Home Smoking Ban, Using Multiply Imputed Data (n = 749), Arizona, January 1, 2011, Through July 26, 2015

Variable	Odds Ratio (95% Confidence Interval)
**Sex**	
Male	1.06 (0.75–1.48)
Female	1 [Reference]
**Age (per 5-y increase in age)^b^**	1.08 (0.99–1.17)
**Race**	
White	1 [Reference]
Black	1.06 (0.62–1.22)
Other	1.75 (0.86–3.58)
**Hispanic or Latino ethnicity**	
Yes	1.55 (0.87–2.76)
No	1 [Reference]
**Insurance type**	
Private	1 [Reference]
Not insured or AHCCCS	0.87 (0.62–1.22)
**Has high school diploma, n (%)**	
Yes	1 [Reference]
No	0.82 (0.51–1.31)
**Child (<18 y) resides in household**	
Yes	0.92 (0.54–1.56)
No	1 [Reference]
**Other smokers reside in household**	
Yes	0.60 (0.43–0.84)
No	1 [Reference]
**Nicotine dependence (per 1-point increase in score)^c^**	0.98 (0.91–1.06)
**Age of first initiation (per 5-y increase in age)^d^**	1.11 (0.95–1.30)
**Has confidence to quit for 24 hours^e^**	
Yes	0.77 (0.48–1.23)
No	1 [Reference]
**Has at least 1 chronic health condition^f^**	
Yes	0.48 (0.32–0.70)
No	1 [Reference]
**Has a mental health condition^g^**	
Yes	0.94 (0.67–1.34)
No	1 [Reference]
**Uses medication for tobacco use cessation**	
Yes	1.16 (0.76–1.79)
No	1 [Reference]
**Number of telephone coaching sessions before 7-month follow-up**	
0–4 calls	1 [Reference]
≥5 calls	3.25 (2.33–4.55)

Abbreviations: AHCCCS, Arizona Health Care Cost Containment System; ASHLine, Arizona Smokers’ Helpline.

a Defined as no use of tobacco in the previous 30 days, reported at 7-month follow-up.

b Odds ratios were calculated for an increment of 5 years in age. That is, for every 5-year increase in age, the odds of quitting tobacco for those who implemented a complete home smoking ban was 1.08 times the odds of those who had not implemented a ban.

c Measured by the Fagerström Test for Nicotine Dependence score (ranging from 0–10), with higher scores indicating greater dependence (12). Odds ratios were calculated for a 1-point increase in the Fagerström Test score.

d Odds ratios were calculated for an increment of 5 years in age.

e Dichotomized as “confident” (caller’s response of “confident,” “very confident,” or “extremely confident”) and “not confident” (responses of “somewhat confident” or “not confident”).

f One or more of the following: asthma, hypertension, cancer, chronic obstructive pulmonary disease, diabetes, or heart disease.

g Treated for any of the following conditions: mental health or emotional challenges, such as anxiety disorder, depression, bipolar disorder, alcohol or drug abuse, or schizophrenia.

## Discussion

To our knowledge, this is the first study to examine factors that predict tobacco use cessation among a subgroup of callers who implemented a complete HSB after enrolling in quitline services. Only 65.4% of callers who implemented HSBs reported a 30-day quit at 7-month follow-up. Thus, callers did not equally benefit from implementing a complete HSB during the quitting process. Callers who implemented a complete HSB and had increased odds of tobacco use cessation at 7-month follow-up were more likely to not have a chronic health condition, to not live with another smoker in the home, and to have participated in more coaching sessions. The identified predictors among callers who implemented complete HSBs were consistent with predictors of cessation among the general population of smokers (4,17–21). For example, a Cochrane review found evidence of a dose-response relationship between the number of coaching sessions and higher quit rates (17). Other studies indicated that having a chronic health condition is associated with a lower likelihood of tobacco use cessation, although cessation rates among quitline users are higher than cessation rates from primary-care–based smoking interventions (20). Previous studies suggested that the absence of other smokers in the home is an important predictor of changes in smoking behavior (4,21) and aids in the implementation of HSBs since households with fewer smokers potentially have fewer barriers to restricting smoking.

Our findings may be particularly valuable for quitlines that want to continue the trend of increasing their breadth of services (22). Quitlines that help callers implement complete HSBs and promote cessation and smoke-free homes support 2 major goals of the Centers for Disease Control and Prevention. The goals are promoting quitting among adults and youths and eliminating exposure to secondhand smoke through the development of comprehensive state plans to decrease tobacco-use rates (7). Our findings suggest that quitlines interested in implementing HSB interventions should develop specialized protocols for callers with particular characteristics to optimize quitline service delivery and increase quit rates. However, tobacco users who have chronic health conditions and/or live with other smokers may also benefit from HSB interventions if quitlines address the unique challenges faced by these tobacco users in implementing and enforcing smoking bans. For example, these tobacco users may need additional skills, strategies, and support to change multiple health behaviors and involve other smokers in the home in their quitting process.

This study had several limitations. One limitation was the lack of available data on HSBs between enrollment and 7-month follow-up. No information was available on when the HSB was implemented during the quitting process, length of implementation, or enforcement of the HSB, all of which may have influenced cessation. Additionally, the study lacked temporal data and was unable to determine if the complete HSB preceded or followed quitting tobacco. To reduce participation burden, callers were not asked to indicate their level of income during enrollment; instead, we used type of health insurance as a proxy for socioeconomic status (23). Another limitation was the use of self-reported data, which may have resulted in recall and social desirability biases; however, the collection of self-reported data is standard practice among quitlines. We also found a substantial amount of missing data at enrollment and 7-month follow-up. However, the initial data set was large, and multiple imputation in the sensitivity analysis generally supported the results of the primary analysis.

Although implementation of HSBs is associated with smoking behaviors and cessation (2,3,24–26), little research exists on the acceptability, feasibility, and effectiveness of interventions to establish smoke-free households among quitline callers. This study suggested that certain groups of quitline callers may be more likely than other groups of callers to quit tobacco use when they implement complete HSBs as part of their quitting process. Expanding quitline services to include HSB interventions may have other advantages, including improving callers’ engagement, motivation, and confidence in quitline services. These advantages could contribute to increased cessation rates among the quitline population.

## References

[1] Pierce J . Evaluating the effectiveness of smoke-free policies. Lyon (FR): International Agency for Research on Cancer Prevention, Tobacco Control; 2009.

[2] Pizacani BA , Martin DP , Stark MJ , Koepsell TD , Thompson B , Diehr P . A prospective study of household smoking bans and subsequent cessation related behaviour: the role of stage of change. Tob Control 2004;13(1):23–8. 10.1136/tc.2003.003038 14985591 PMC1747832

[3] Zablocki RW , Edland SD , Myers MG , Strong DR , Hofstetter CR , Al-Delaimy WK . Smoking ban policies and their influence on smoking behaviors among current California smokers: a population-based study. Prev Med 2014;59:73–8. 10.1016/j.ypmed.2013.11.018 24291748

[4] Yong LC , Luckhaupt SE , Li J , Calvert GM . Quit interest, quit attempt and recent cigarette smoking cessation in the US working population, 2010. Occup Environ Med 2014;71(6):405–14. 10.1136/oemed-2013-101852 24497440 PMC4528304

[5] Rees VW , Keske RR , Blaine K , Aronstein D , Gandelman E , Lora V , Factors influencing adoption of and adherence to indoor smoking bans among health disparity communities. Am J Public Health 2014;104(10):1928–34. 10.2105/AJPH.2013.301735 25208003 PMC4160813

[6] Kegler MC , Haardӧrfer R , Berg C , Escoffery C , Bundy L , Williams R , Challenges in enforcing home smoking rules in a low-income population: implications for measurement and intervention design. Nicotine Tob Res 2016;18(5):976–81. 10.1093/ntr/ntv165 26246049 PMC5444099

[7] Centers for Disease Control and Prevention. Best practices for comprehensive tobacco control programs — 2014. Atlanta (GA): US Department of Health and Human Services, Centers for Disease Control and Prevention, National Center for Chronic Disease Prevention and Health Promotion, Office on Smoking and Health; 2014.

[8] Centers for Disease Control and Prevention. Telephone quitlines: a resource for development, implementation, and evaluation. Atlanta (GA): US Department of Health and Human Services, Centers for Disease Control and Prevention, National Center for Chronic Disease Prevention and Health Promotion, Office on Smoking and Health; 2004.

[9] Caponnetto P , Polosa R . Common predictors of smoking cessation in clinical practice. Respir Med 2008;102(8):1182–92. 10.1016/j.rmed.2008.02.017 18586479

[10] Anderson CM , Zhu SH . Tobacco quitlines: looking back and looking ahead. Tob Control 2007;16(Suppl 1):i81–6. 10.1136/tc.2007.020701 18048638 PMC2598521

[11] Fildes EE , Wilson MA , Crawford BJ , Kapella-Mshigeni S , Wilson LA , Henkelman W . Tobacco quitlines in the United States. Nurs Clin North Am 2012;47(1):97–107. 10.1016/j.cnur.2011.10.009 22289401

[12] Heatherton TF , Kozlowski LT , Frecker RC , Fagerström KO . The Fagerström Test for Nicotine Dependence: a revision of the Fagerström Tolerance Questionnaire. Br J Addict 1991;86(9):1119–27. 10.1111/j.1360-0443.1991.tb01879.x 1932883

[13] North American Quitline Consortium. Quitline service offering models: a review of the evidence and recommendations for practice in times of limited resources. Phoenix (AZ): North American Quitline Consortium; 2012.

[14] Azur MJ , Stuart EA , Frangakis C , Leaf PJ . Multiple imputation by chained equations: what is it and how does it work? Int J Methods Psychiatr Res 2011;20(1):40–9. 10.1002/mpr.329 21499542 PMC3074241

[15] Rubin DB . Multiple imputation for nonresponse in surveys. New York (NY): Wiley; 1987.

[16] Desquilbet L , Mariotti F . Dose-response analyses using restricted cubic spline functions in public health research. Stat Med 2010;29(9):1037–57. 20087875 10.1002/sim.3841

[17] Stead LF , Hartmann-Boyce J , Perera R , Lancaster T . Telephone counselling for smoking cessation. Cochrane Database Syst Rev 2013(8):CD002850. 10.1002/14651858.CD002850.pub3 23934971

[18] Hyland A , Borland R , Li Q , Yong HH , McNeill A , Fong GT , Individual-level predictors of cessation behaviours among participants in the International Tobacco Control (ITC) Four Country Survey. Tob Control 2006;15(Suppl 3):iii83–94. 10.1136/tc.2005.013516 16754952 PMC2593055

[19] Gilbert H , Sutton S . Evaluating the effectiveness of proactive telephone counselling for smoking cessation in a randomized controlled trial. Addiction 2006;101(4):590–8. 10.1111/j.1360-0443.2006.01398.x 16548938

[20] Bush T , Zbikowski SM , Mahoney L , Deprey M , Mowery P , Cerutti B . State quitlines and cessation patterns among adults with selected chronic diseases in 15 states, 2005–2008. Prev Chronic Dis 2012;9:E163. 10.5888/pcd9.120105 23137862 PMC3498947

[21] Vangeli E , Stapleton J , Smit ES , Borland R , West R . Predictors of attempts to stop smoking and their success in adult general population samples: a systematic review. Addiction 2011;106(12):2110–21. 10.1111/j.1360-0443.2011.03565.x 21752135

[22] McAfee TA . Quitlines a tool for research and dissemination of evidence-based cessation practices. Am J Prev Med 2007;33(6, Suppl):S357–67. 10.1016/j.amepre.2007.09.011 18021911

[23] National Research Council (US) Panel on DHHS Collection of Race and Ethnic Data. Eliminating health disparities: measurement and data needs. Ver Ploeg M, Perrin E, editors. Washington (DC): National Academies Press; 2004. 25009872

[24] Mills AL , Messer K , Gilpin EA , Pierce JP . The effect of smoke-free homes on adult smoking behavior: a review. Nicotine Tob Res 2009;11(10):1131–41. 10.1093/ntr/ntp122 19633273

[25] Borland R , Yong HH , Cummings KM , Hyland A , Anderson S , Fong GT . Determinants and consequences of smoke-free homes: findings from the International Tobacco Control (ITC) Four Country Survey. Tob Control 2006;15(Suppl 3):iii42–50. 16754946 10.1136/tc.2005.012492 PMC2593064

[26] Hyland A , Higbee C , Travers MJ , Van Deusen A , Bansal-Travers M , King B , Smoke-free homes and smoking cessation and relapse in a longitudinal population of adults. Nicotine Tob Res 2009;11(6):614–8. 10.1093/ntr/ntp022 19346505 PMC2722236

